# Intensivmedizinische Versorgung von Patienten mit akuter Intoxikation in Deutschland – ein Rückblick über 20 Jahre: Akute Vergiftungen auf der Intensivstation

**DOI:** 10.1007/s00063-022-00937-1

**Published:** 2022-06-27

**Authors:** Kristin Bremen, Theresa H. Wirtz, Jonathan F. Brozat, Samira Abu Jhaisha, Philipp Hohlstein, Maike Pollmanns, Lukas Buendgens, Christian Trautwein, Alexander Koch

**Affiliations:** Medizinische Klinik III, Uniklinik RWTH Aachen, Pauwelsstr. 30, 52074 Aachen, Deutschland

**Keywords:** Vergiftungen, Antidepressiva, Suizid, Benzodiazepine, Organversagen, Drug overdose, Antidepressants, Suicide, Benzodiazepines, Organ failure

## Abstract

**Hintergrund:**

Die vorliegende Studie präsentiert ein Kollektiv akut intoxikierter Patienten, die in den letzten 2 Jahrzehnten auf eine medizinische Intensivstation eines tertiären Versorgungszentrums in Deutschland aufgenommen wurden.

**Ziel der Arbeit:**

Das Ziel der Studie bestand darin, einen Überblick zur akuten Intoxikation als relevantes intensivmedizinisches Krankheitsbild und hiermit assoziierte klinische Charakteristika sowie die Prognose der betroffenen Patienten zu bieten.

**Material und Methoden:**

Die Studienkohorte umfasst 1030 Patienten, die in den Jahren1999–2019 aufgrund einer akuten Vergiftung auf die medizinische Intensivstation der Uniklinik RWTH Aachen aufgenommen wurden. Demographische und klinische Merkmale sowie das klinische Management wurden detailliert analysiert und zwischen alters- und geschlechtsspezifischen Untergruppen verglichen.

**Ergebnisse:**

Suizidversuche stellten die häufigste Ursache für die intensivmedizinische Aufnahme intoxikierter Patienten dar. Insbesondere führten Medikamente, v. a. Antidepressiva, zu einer akuten Vergiftung. Die Substanzen variierten hierbei je nach Geschlecht und Alter der betroffenen Patienten. In der Subgruppe der älteren Patienten stellten Benzodiazepine die am häufigsten verwendeten Substanzen dar. 286 Patienten (28 %) entwickelten ≥ 1 Organversagen. Die Gesamtmortalität betrug 2,6 %. Im Vergleich der ersten (1999–2009) mit der zweiten Dekade (2010–2019) des Beobachtungszeitraums zeigte sich ein Trend zu einer häufigeren Einnahme von Antidepressiva und Alkohol, während die Verwendung von Benzodiazepinen rückläufig war.

**Diskussion:**

Obwohl die Gesamtmortalität im beobachteten Kollektiv gering ist, repräsentieren akut vergiftete Patienten fast 10 % aller Einweisungen auf die Intensivstation und beanspruchen insbesondere in Zeiten begrenzter Intensivkapazitäten wertvolle Ressourcen.

## Abstract (en)

**Background:**

This study presents a relevant collective of patients with acute poisoning admitted to a medical intensive care unit (ICU) of a tertiary care center in Germany during the past two decades.

**Objectives:**

Our study aims at providing an overview of patients’ motivation for intoxication, their behavioral patterns, and subsequent treatment regimens.

**Materials and methods:**

A total of 1030 patients admitted to the medical ICU of the RWTH Aachen University Hospital due to acute poisoning from January 1999 to December 2019 were included in our study. Demographic and clinical characteristics, as well as therapeutic procedures and outcomes were analyzed in detail and compared between age- and gender-related subgroups.

**Results:**

The most common substances that led to acute poisoning were medical substances, especially antidepressants. Substances varied between gender and age. The most frequent cause leading to acute poisoning in both females and males was suicidal intent associated with an intake of antidepressants. 286 patients (28%) developed ≥ 1 organ failure. The overall mortality was 2.6%. Comparing the first (1999–2009) and second decade (2010–2019), there was a distinct trend towards a more frequent intake of antidepressants and alcohol, whereas the use of benzodiazepines decreased.

**Conclusions:**

Although the overall patient mortality was low, patients with acute poisoning accounted for nearly 10% of all ICU admissions and required valuable resources despite limited capacity.

Akute Vergiftungen stellen eine häufige Indikation für eine intensivmedizinische Behandlung dar. Der klinische Verlauf betroffener Patienten variiert in Abhängigkeit von der eingenommenen Substanz und deren Dosis sowie klinischer und demographischer Charakteristika des betroffenen Patienten als auch der Ursache der Vergiftung. Patienten mit akuten Vergiftungen repräsentieren sowohl national als auch international ein relevantes Kollektiv im intensivstationären Bereich, wenn auch die Mortalität gering ist. Mit unserem Beitrag bieten wir einen beispielhaften Überblick über klinische Charakteristika der Behandlung akut intoxikierter Patienten auf einer medizinischen Intensivstation in Deutschland im Verlauf der letzten 20 Jahre.

## Einleitung

Vergiftungen, Überdosierungen und selbstverletzendes Verhalten stellen nach wie vor ein relevantes Problem auf den Intensivstationen in aller Welt dar [3, 10, 11]. Eine Intoxikation geschieht hierbei in den meisten Fällen im Kontext einer suizidalen Absicht des betroffenen Patienten [12]. Die Häufigkeit der für die Intoxikation verwendeten Substanzen ist abhängig von der geographischen Lage: Antidepressiva, Alkohol und Benzodiazepine sind in Westeuropa die am häufigsten verwendeten Substanzen, während in Asien unter anderem Pestizide zu den vielfach gebrauchten Substanzen gehören [2, 13, 23].

Vergiftete Patienten repräsentieren in westlichen Ländern 3–6 % aller Intensiveinweisungen [11, 17]. Eine Intoxikation hat dabei nicht nur Auswirkungen auf den Gesundheitszustand der Patienten: Auch die erforderlichen Behandlungsmaßnahmen auf der Intensivstation verbrauchen wertvolle personelle und materielle Ressourcen.

Unsere Arbeit präsentiert eine 20-jährige retrospektive Beobachtungsstudie, die akut vergiftete Patienten und deren intensivmedizinisches Management analysiert. Die Untersuchungen umfassen demographische Daten mit Schwerpunkt auf Veränderungen im Konsumverhalten über die Zeit sowie Auffälligkeiten im klinischen Verlauf der Patienten.

## Methoden

### Studiendesign, Umfang und Setting

Unsere Analyse umfasst insgesamt 1030 Patienten, die zwischen Januar 1999 und Dezember 2019 aufgrund einer akuten Vergiftung auf die medizinische Intensivstation der Uniklinik RWTH Aachen aufgenommen wurden. Die volljährigen Patienten erfüllten die Diagnose einer akuten Vergiftung oder eines Substanzmissbrauchs, die eine intensivmedizinische Überwachung und Behandlung erforderten. Die Identifizierung der Studienpatienten erfolgte retrospektiv durch die Anwendung von Diagnoseschlüsseln aus dem Katalog der internationalen statistischen Klassifikation der Krankheiten und verwandter Gesundheitsprobleme (ICD-10, „international statistical classification of diseases and related health problems“). Es wurden ICD-10-Codes verwendet, die sich auf Substanzmissbrauch oder akute Vergiftungen beziehen (Tab. 1).

**Table 1:** 

ICD-Code	Bezeichnung
*F10.0*	Psychische Störungen und Verhaltensstörungen aufgrund von *Alkoholkonsum*
*F11.0*	Psychische Störungen und Verhaltensstörungen aufgrund von *Opioiden*
*F12.0*	Psychische Störungen und Verhaltensstörungen aufgrund von *Cannabinoiden*
*F13.0*	Psychische Störungen und Verhaltensstörungen aufgrund von *Sedativa* und *Hypnotika*
*F14.0*	Psychische Störungen und Verhaltensstörungen aufgrund von *Kokain*
*F15.0*	Psychische Störungen und Verhaltensstörungen aufgrund von *Stimulanzien*
*F16.0*	Psychische Störungen und Verhaltensstörungen aufgrund von *Halluzinogenen*
*F19.0*	Psychische Störungen und Verhaltensstörungen aufgrund von *multiplem Drogengebrauch*
*F55.–*	Missbrauch von nichtabhängigkeitserzeugenden Substanzen
*T39.–*	Vergiftungen durch nichtopioide *Analgetika, Antipyretika und Antirheumatika*
*T40.–*	Vergiftungen durch *Narkotika und Psycholeptika*
*T42.–*	Vergiftungen durch *Antiepileptika, Sedativa, Hypnotika* und *Anti-Parkinson-Mittel*
*T43.–*	Vergiftungen durch *Psychopharmaka*, nicht anderweitig klassifiziert
*T51.–*	Toxische Wirkung von *Alkohol*
*T58.–*	Toxische Wirkung von *Kohlenstoffmonoxid*
*T60.–*	Toxische Wirkung von *Pestiziden*
*T62.–*	Toxische Wirkung anderer *schädlicher Stoffe*, die mit der Nahrung aufgenommen werden
*T65.–*	Toxische Wirkung von anderen und *nichtspezifizierten Stoffen*

Es wurden nur diejenigen Patienten in unsere Studie eingeschlossen, die aufgrund einer Vergiftung auf der Intensivstation behandelt werden mussten. Patienten mit dem Nebenbefund einer Intoxikation wurden nicht mit ins Kollektiv aufgenommen.

### Datenquellen und Patientenauswahl

Für die gesamte Studiengruppe standen folgende Parameter zur Verfügung: eingenommene Substanz, Alter, Geschlecht, Hauptdiagnose bei Aufnahme, Anamnese zu Substanzmissbrauch oder psychiatrischen Störungen, Aufnahmedatum, Entlassungsdatum, die Notwendigkeit einer mechanischen Beatmung oder Hämodialyse, Todesdatum und Todesursache. Die Vergiftung wurde als „Mischintoxikation“ beschrieben, wenn bei der Einnahme mehrerer Substanzen keine Leitsubstanz identifiziert werden konnte. Alle verfügbaren Parameter wurden in die Analyse einbezogen.

Die demographischen Merkmale des Studienkollektivs wurden mit den Merkmalen eines allgemeinen ICU-Kollektivs kritisch kranker Patienten verglichen. Hierzu bedienten wir uns bereits vorliegender Analysen unseres ICU-Kollektivs, die jedoch in dem vorliegenden Manuskript nicht im Detail dargestellt werden sollen.

### Statistische Methoden

Die Daten wurden deskriptiv erhoben und in absoluten oder relativen Zahlen bezogen auf die jeweilige Subgruppe der Patienten angegeben. Zur Bewertung der statistischen Signifikanz zwischen den einzelnen Gruppen wurden der χ^2^-Test von Pearson und *Fisher’s-exact-*Test verwendet [5, 16]. Ein *p*-Wert von < 0,05 wurde als statistisch signifikant angesehen (*p < 0,05; **p < 0,01; ***p < 0,001). Alle statistischen Analysen wurden mit SPSS, Version 25, (SPSS, Chicago, IL, USA) durchgeführt.

## Ergebnisse

### Charakteristika der Studienkohorte

Im Studienzeitraum von 1999–2019 wurden insgesamt 13.213 Patienten auf der Intensivstation der Uniklinik RWTH Aachen aufgenommen – 1030 (7,8 %) aufgrund einer akuten Vergiftung. Das Durchschnittsalter der eingeschlossenen Patienten betrug 40 Jahre und lag damit unter dem Durchschnittsalter des ICU-Gesamtkollektivs (61 Jahre), das zum Vergleich herangezogen wurde. Im Gegensatz zu einem Anteil von nur 39 % weiblichen Patientinnen im ICU-Vergleichskollektiv waren in unserem Intoxikationskollektiv 54 % (*n* = 558) Frauen. In den 4 definierten Altersgruppen 31–50, 51–70 und > 71 Jahre waren die Geschlechter gleich verteilt; einzig in der untersten Altersgruppe 18–30 Jahre überwog der Anteil weiblicher Patienten (Tab. 2). In den meisten Fällen (*n* = 690; 67 %) kam es zu Vergiftungen durch medizinische Substanzen: Innerhalb dieser Subgruppe waren Antidepressiva am häufigsten (*n* = 175; 17 %). Benzodiazepine wurden in etwa 12 % und Opioide in 10 % aller Fälle dokumentiert. Neben medizinischen Substanzen führte Alkohol in 14 % der Fälle zu einer akuten Intoxikation. Pilze und Kohlenmonoxid wurden bei weniger als 3 % aller eingeschlossenen Patienten angegeben. Interessanterweise wurde bei jedem 10. Patienten eine Mischintoxikation diagnostiziert. Weitere detaillierte Patientenmerkmale und Substanzen sind in Tab. 2 aufgeführt.

**Table 2:** 

Parameter	Patienten
**Alter, *n* (Altersspanne)**	40 (18–92)
*18–30 Jahre, n (%)*	326 (31,6)
Weiblich, *n* (%)	207 (63,5)***
*31–50 Jahre, n (%)*	459 (44,6)
Weiblich, *n* (%)	224 (48,8), n. s.
*51–70 Jahre, n (%)*	187 (18,2)
Weiblich, *n* (%)	99 (52,9), n. s.
*>* *71 Jahre, n (%)*	58 (5,6)
Weiblich, *n* (%)	28 (48,3), n. s.
**Geschlecht, *n*** **(%)**	
*Männlich*	472 (45,8)
*Weiblich*	558 (54,2)
**Substanz, *n*** **(%)**	
*Medizinische Substanzen*	690 (67,0)
Antidepressiva	175 (17,0)
Benzodiazepine	120 (11,7)
Opiate	102 (9,9)
Paracetamol	86 (8,3)
Antipsychotika	78 (7,6)
Sedativa, Hypnotika, Antikonvulsiva	59 (5,7)
NSAR	34 (3,3)
Medizinische Substanzen < 3 %	36 (3,5)
*Alkohol*	148 (14,4)
*Andere Substanzen*	88 (8,6)
Kohlenstoffmonoxid	11 (1,1)
Pilze	11 (1,1)
Tabak	8 (0,8)
Andere Noxen	58 (5,6)
*Mischintoxikationen*	104 (10,1)

Die Daten sind nach Verfügbarkeit angegeben. Unter „medizinische Substanzen < 3 %“ werden verschiedene Substanzen bzw. Substanzgruppen (z. B. kardiologische Medikamente, Lithium) subsummiert, die im Vergleich zu den anderen aufgeführten Substanzen jeweils nur bei einem Anteil von < 3 % aller Vergiftungen ursächlich waren. ***p < 0,001

*NSAR* nichtsteroidales Antirheumatikum

### Benzodiazepineinnahme häufiger bei älteren Patienten

**Figure 1:**
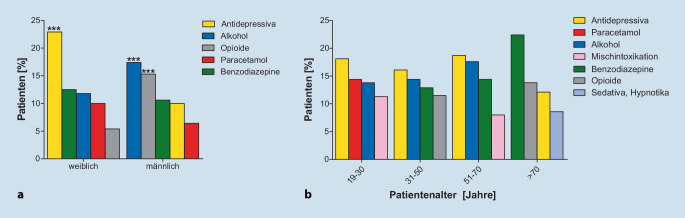

Im Geschlechtervergleich traten bei weiblichen Patienten akute Vergiftungen am häufigsten durch Antidepressiva auf (*n* = 128; 23 %). In der Gruppe der vergifteten männlichen Patienten waren hingegen Vergiftungen mit Alkohol (*n* = 82; 17 %), Opioiden (*n* = 72; 15 %) oder Benzodiazepinen (*n* = 50; 11 %) – in Abgrenzung zu den sonstigen Sedativa, die als „Sedativa, Hypnotika und Antidepressiva“ subsummiert wurden – am häufigsten (Abb. 1a).

Über mehrere Altersgruppen hinweg stellten Antidepressiva die am häufigsten verwendete Substanz dar (19–30 J.: *n* = 59; 18 %; 31–50 J.: *n* = 74; 16 %; 51–70 J.: *n* = 35; 19 %). Lediglich bei Patienten ≥ 71 Jahre erwiesen sich Antidepressiva nur als dritthäufigste Substanz. Benzodiazepine wurden in der ältesten Altersgruppe am häufigsten verwendet (*n* = 13; 24 %). Interessanterweise war die einzige Gruppe, die eine auffallend hohe Rate an Paracetamol (*n* = 47; 14,4 %) aufwies, die jüngste Altersgruppe zwischen 19 und 30 Jahren (Abb. 1b).

### Psychiatrische Störungen prädisponieren für akute Vergiftungen

Anschließend untersuchten wir, ob bei Patienten, die aufgrund einer Vergiftung aufgenommen wurden, vorbestehende psychiatrische Störungen festgestellt werden konnten. Interessanterweise wurde bei zwei Dritteln (*n* = 529; 69,2 %) bereits im Vorfeld oder während des Aufenthalts eine psychiatrische Erkrankung diagnostiziert, während bei fast einem Fünftel (21 %) mehr als eine psychiatrische Störung diagnostiziert wurde (Tab. 3). Depressionen waren die am häufigsten vorkommende psychiatrische Diagnose (*n* = 200; 26,2 %). In Bezug auf die Ursache der Vergiftung war in den meisten Fällen eine Selbstmordabsicht erkennbar (*n* = 520; 68,1 %). Nur ein kleiner Teil der Patienten litt an einer versehentlichen Vergiftung (*n* = 56; 7,3 %).

**Table 3:** 

Psychiatrische Diagnosen	
*Vorbestehende psychiatrische Diagnose, n (%)*	529 (69,2)
Patienten mit ≥ 1 psychiatrischer Diagnose	160 (21,0)
Depression	200 (26,2)
Alkoholmissbrauch	141 (18,5)
*Ursache der Vergiftung, n (%)*	
Suizidalität	520 (68,1)
Substanzmissbrauch	67 (8,8)
Versehentlich	56 (7,3)

### Antidepressiva am häufigsten im Rahmen eines Suizidversuchs verwendet

**Figure 2:**
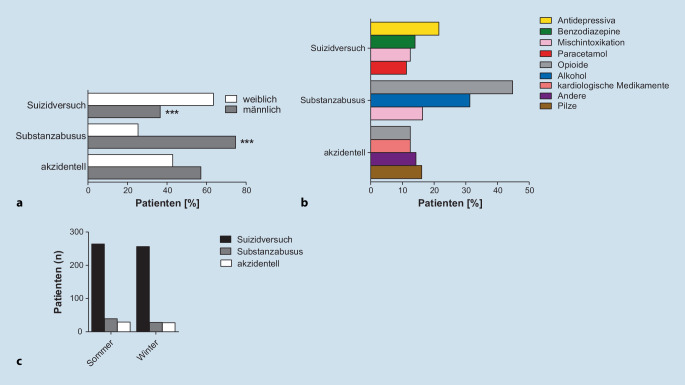

In der Patientengruppe, die einen Suizidversuch unternahm, waren deutlich mehr Patienten weiblich als männlich (63 % gegenüber 37 %, *p* ≤ 0,001; Abb. 2a). Zweithäufigste Ursache für akute Vergiftungen war der Substanzmissbrauch. In dieser Untergruppe war der Anteil der männlichen Patienten signifikant höher (75 % vs. 25 %; *p* ≤ 0,001). In der Untergruppe der Patienten mit Suizidabsichten wurden am häufigsten Antidepressiva (*n* = 112; 21,5 %) und Benzodiazepine (*n* = 75; 14,4 %) verwendet, während Paracetamol nur in 11 % (*n* = 59) eingesetzt wurde. Im Gegensatz dazu waren innerhalb der Kategorie Substanzmissbrauch die häufigsten Substanzen Opioide (*n* = 30; 44,8 %) bzw. Alkohol (*n* = 21; 31,3 %). Bei Patienten, die sich versehentlich selbst vergifteten, stellten wir Pilze (*n* = 9; 16,1 %) sowie kardiologische Medikamente einschließlich Herzglykosiden und Kalziumkanalblockern fest (*n* = 7; 12,5 %) (Abb. 2b). Pilze werden meist aus Unwissenheit von Pilzsammlern verzehrt oder, in unserem Kollektiv weitaus seltener, als psychoaktive Droge konsumiert. Generell sind Vergiftungen mit Pilzen im klinischen Alltag selten. Außerdem sind Pilzvergiftungen in Deutschland nicht meldepflichtig, sodass eine hohe Dunkelziffer an nichterfassten Fällen nicht auszuschließen ist.

Darüber hinaus stellten wir die Hypothese auf, dass sich die Gründe für akute Vergiftungen in der Sommer- und Wintersaison unterscheiden könnten. In der Sommersaison (April bis Oktober) als auch in der Wintersaison (November bis März) waren die Fälle aufgrund von Selbstmordabsichten, Substanzmissbrauch oder versehentlichen Vergiftungen jedoch gleich verteilt (Abb. 2c). In einer deutschen Veröffentlichung des Statistischen Bundesamtes von 2007 wurden Unterschiede zwischen den Jahreszeiten in Bezug auf vollendete Suizide analysiert [18]. Übereinstimmend mit unseren Ergebnissen zu versuchten Suiziden wurde die Vermutung verworfen, dass sich Menschen vermehrt in den Wintermonaten das Leben nehmen. Beim Vergleich der vollendeten Suizide aus der Destatis-Studie mit unserem Kollektiv fiel auf, dass in der zitierten Studie die männlichen Patienten überwiegen, während bei den versuchten Suiziden in unserem Kollektiv die weiblichen Patienten die Mehrheit darstellen.

### Geringe Prävalenz des (Multi‑)Organversagens

**Figure 3:**
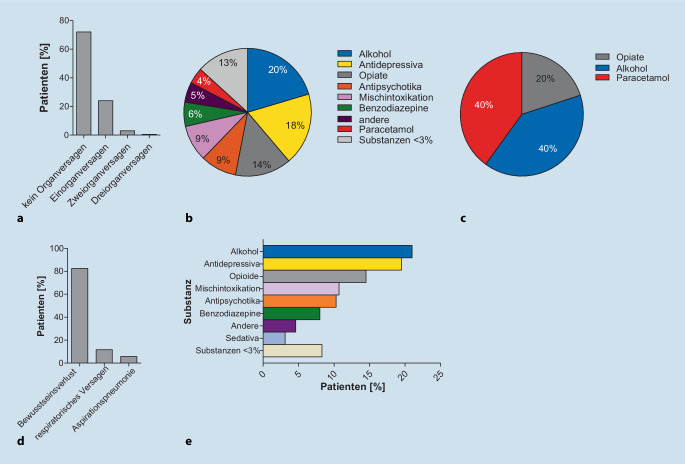

In unserer Kohorte kam es bei den meisten der beobachteten Patienten zu keinerlei Organversagen (*n* = 744; 72,2 %; Abb. 3a). Allerdings erlitten 248 Patienten (24,1 %) ein Organversagen mindestens eines Organs, das entweder ein Atemwegs‑, Nieren- oder Leberversagen einschloss. 33 Patienten (3,2 %) erlitten ein Zweiorganversagen und nur 5 Patienten (0,5 %) wiesen ein Dreiorganversagen auf (Abb. 3a).

Bei den Patienten, die unter einem Organversagen mindestens eines Organs litten, war Alkohol in 57 Fällen (19,9 %) die führende Substanz, gefolgt von Antidepressiva in 52 Fällen (18,2 %). Nur 11 Patienten (3,8 %) erlitten ein Organversagen aufgrund einer Paracetamolvergiftung (Abb. 3b). Innerhalb der kleinen Gruppe von 5 Patienten, die ein Dreiorganversagen entwickelten, wurden jedoch jeweils 2 Patienten durch Paracetamol oder Alkohol vergiftet (Abb. 3c).

### Akute Intoxikation: ein Patientenkollektiv mit geringer Mortalität

**Figure 4:**
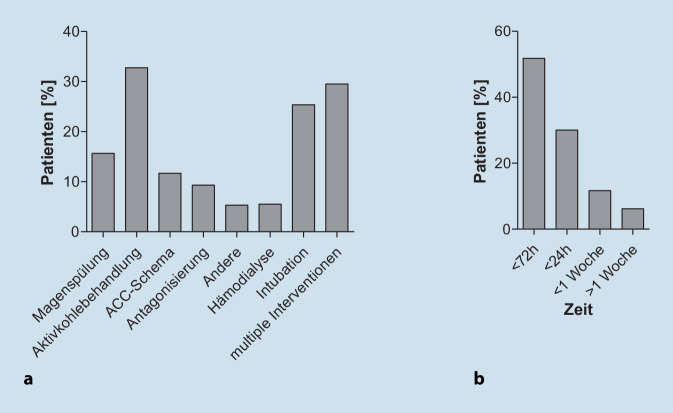

Bei 225 Patienten (29,5 %) wurden mehrere Interventionen durchgeführt. Die häufigste Intervention war die Gabe von Aktivkohle (*n* = 251; 32,6 %). Eine Intubation war bei 262 Patienten (25,4 %) erforderlich; eine Hämodialyse wurde bei 57 Patienten (5,5 %) notwendig. Der Hauptgrund für die Intubation war ein Bewusstseinsverlust gefolgt von primärem respiratorischem Versagen (Abb. 3d). Die meisten Patienten, die mechanisch beatmet werden mussten, wiesen eine akute Vergiftung aufgrund von Alkoholkonsum auf (Abb. 3e). Zu den zusätzlichen Interventionsmaßnahmen gehörten Magenspülung (*n* = 120; 15,7 %), Behandlung mit N‑Acetylcystein (NAC; *n* = 89; 11,7 %) oder Behandlung mit Antagonisten (*n* = 71; 9,3 %; Abb. 4a).

Die Dauer des Aufenthalts jedes Patienten auf der Intensivstation richtete sich nach dem Schweregrad der Vergiftung. Die meisten Patienten verbrachten weniger als 72 h auf der Intensivstation (*n* = 534; 51,8 %; Abb. 4b). Die Substanzen, die zu einer Aufenthaltsdauer von mehr als einer Woche führten, waren am häufigsten Alkohol, Opioide (jeweils *n* = 12; 18,8 %) und Benzodiazepine (*n* = 8; 12,5 %).

Insgesamt war die Gesamtsterblichkeit in der beobachteten Kohorte gering (*n* = 27; 2,6 %).

### Am häufigsten verwendete Substanzen: früher Benzodiazepine, heute Antidepressiva

**Figure 5:**
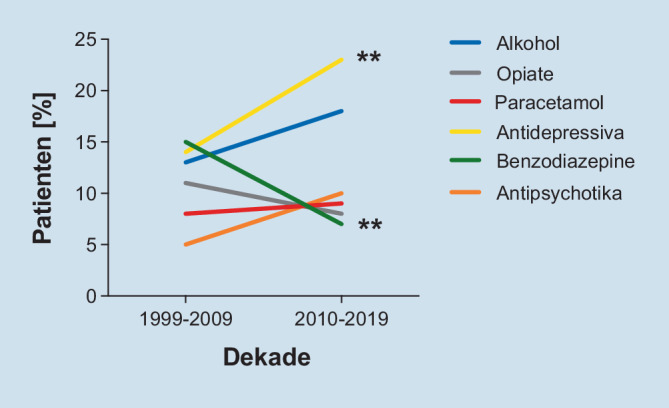

In der ersten Dekade (1999–2009) repräsentierten Benzodiazepine die am häufigsten konsumierten Substanzen, die zu einem Aufenthalt auf der Intensivstation führten (*n* = 89; 15,3 %). Die zweithäufigsten Substanzen waren Antidepressiva (*n* = 79; 13,6 %), gefolgt von Alkohol (*n* = 69; 11,9 %). Antipsychotika machten in der ersten Dekade 5 % (*n* = 30) aus (Abb. 5a). In der zweiten Dekade (2010–2019) hingegen stellten Antipsychotika die dritthäufigste konsumierte Substanz dar (*n* = 48; 10,7 %). Die häufigsten konsumierten Substanzen in der zweiten Dekade waren Antidepressiva (*n* = 96; 21,3 %), gefolgt von Alkohol (*n* = 79; 17,6 %). Benzodiazepine führten nur zu ca. 7 % der Intoxikationen innerhalb der zweiten Dekade (Abb. 5b).

## Diskussion

Die in unsere Studie eingeschlossenen, akut intoxikierten Patienten wiesen ein breites Spektrum an verwendeten Substanzen auf. Die dargestellten Häufigkeiten der Substanzen sind vergleichbar mit den Ergebnissen anderer europäischen Studien [2, 6, 12]. Darüber hinaus steht die Assoziation der verwendeten Substanzen mit dem Geschlecht und Alter des Patienten sowie dem Motiv der Vergiftung im Einklang mit einer vergleichbaren deutschen Studie [20]. So gehörten beispielsweise Antidepressiva in jeder Altersgruppe zu den am häufigsten konsumierten Substanzen. Als nächstes zeigten Paracetamol und Benzodiazepine ein häufiges Vorkommen in der jüngsten bzw. ältesten Altersgruppe. Dies steht im Einklang mit Huang et al., die einen Anstieg der Paracetamolvergiftungen im jüngeren Drittel ihrer Kohorte feststellten [7]. Die internationalen Unterschiede bei Substanzen, die zu akuten Vergiftungen führen, sind auffallend variierend, insbesondere wenn man den asiatischen und den europäischen Kontinent vergleicht: So gehört Kohlenmonoxid (CO) in einer Studie aus Hongkong zu den 4 häufigsten Substanzen, während in unserer Studie nur bei 11 Patienten (1,1 %) eine Vergiftung durch CO festgestellt wurde [10]. Dies könnte zum einen an der erhöhten medialen Berichterstattung über CO-assoziierte Suizidversuche, zum anderen aber auch an den weniger strengen Auflagen zur Luftreinhaltung im asiatischen Raum liegen, die infolge Müllverbrennungen und ungeschützten Kochnischen passiert [10]. In Deutschland hingegen werden Luftreinhaltepläne durch Richtlinien vorgegeben. Zusätzlich waren Vergiftungen mit Pestiziden, die in unserer Kohorte selten auftraten, im Nahen Osten und in China von größerer Bedeutung [8, 13, 23]. Ursächlich für den hohen Stellenwert der Pestizide in Asien könnten die einfachere Verfügbarkeit, der extensive und unsichere Gebrauch in der Landwirtschaft sowie die unkontrollierte Lagerung zu Hause sein [8, 10]. In Deutschland hingegen unterliegen beispielsweise Pflanzenschutzmittel strengen Regulationen des Bundesamtes für Verbraucherschutz und Lebensmittelsicherheit und sind somit weniger breit verfügbar [15]. Wir schlussfolgern, dass Intoxikationen mit Pestiziden für medizinisches Personal in europäischen Ländern weniger relevant sind [1, 14].

Es ist bekannt, dass Suizidalität ein herausragendes Motiv für Vergiftungen darstellt und mit dem weiblichen Geschlecht assoziiert ist. Mit unserer Studie und in Übereinstimmung mit einer kürzlich veröffentlichten deutschen Studie zeigen wir, dass sich dieser Aspekt in den letzten Jahren nicht verändert hat und dass die Motivation für Vergiftungen aufgrund von Suizidabsichten bei Frauen immer noch deutlich höher ist als bei Männern [20]. Darüber hinaus konnten wir Suizidalität als ganzjähriges und nicht nur saisonales Problem charakterisieren, was abermals die Relevanz akuter Vergiftungen im intensivstationären Bereich unterstreicht.

Bei 5 der eingeschlossenen Patienten kam es zu einem Dreiorganversagen. Dabei wurden je 2 der Dreiorganversagen durch Alkohol und Paracetamol verursacht. Bei insgesamt 248 Patienten kam es zu einem Organversagen mindestens eines Organs, das in 18 % durch Alkohol ausgelöst wurde. Dieses Ergebnis muss sorgfältig betrachtet werden, da Brandenburg et al. paradoxerweise feststellen, dass eine Alkoholintoxikation eine wahrscheinlich nicht notwendige Behandlung auf der Intensivstation vorhersagen könnte [3]. Bezüglich schwerer Verläufe nach Alkoholintoxikation umfasst unsere Studie nur 5 Patienten mit einem Dreiorganversagen; 2 davon nach Alkoholintoxikation, in 4 von 5 Fällen bestand anamnestisch ein vorbestehender schädlicher Alkoholkonsum. Unsere Hypothese ist, dass insbesondere Patienten mit chronischem Alkoholkonsum einem gesteigerten Risiko eines Mehrorganversagens ausgesetzt sind, jedoch vermag unsere Studie hierzu aufgrund der geringen Patientenanzahl mit höhergradigem, alkoholassoziiertem Organversagen keine generelle Aussage zu treffen.

Im Gegensatz zur Studie von Siedler et al. war der Einsatz von Aktivkohle (ACT) als Interventionsmaßnahme in unserer Patientenkohorte 3‑mal höher [20]. Mit Aktivkohle können u. a. Antidepressiva, Benzodiazepine, NSAR und Paracetamol adsorbiert werden, die in beiden Kohorten zu den am häufigsten verwendeten Substanzen gehören [9]. Die Applikation von Aktivkohle gehört zu den Maßnahmen der primären Giftelimination. Faktoren, die die Gabe von Aktivkohle indizieren, sind unter anderem ein gesicherter Atemweg, eine zu erwartende schwere Toxizität, kürzlich eingenommene Medikamente, Substanzen, von denen bekannt ist, dass sie an Aktivkohle adsorbieren, und das Fehlen eines spezifischen Antidots [4]. Der Einsatz der Aktivkohle ist damit abhängig von der betreffenden Substanz sowie der Einnahmelatenz, was eine verschiedene Häufigkeit der Aktivkohle in verschiedenen Intoxikationskollektiven erklären kann. Die Latenz zwischen Intoxikation und intensivstationärer Aufnahme wird weder in unserer noch der zitierten Studie adressiert und kann bereits den unterschiedlich häufigen Einsatz der Aktivkohle erklären, ebenso ein unterschiedliches Verteilungsmuster der zugrundeliegenden Substanzen. Auch der Einsatz von Antidoten unterschied sich beim Vergleich beider deutschen Studien erheblich. Dieser Befund zeigt wichtige nicht nur internationale, sondern auch nationale Unterschiede im Hinblick auf die Indikationsstellung und Durchführung unterschiedlicher therapeutischer Strategien bei der Behandlung von Intoxikationspatienten auf.

Beim Vergleich der Gesamtzahl der Vergiftungsfälle von 1999–2009 und von 2010–2019 zeigte sich ein deutlicher Anstieg des Konsums von Antipsychotika, Antidepressiva und Alkohol. Benzodiazepinintoxikationen hingegen waren in unserer Kohorte in der 2. Dekade seltener als in der 1., was im Gegensatz zu früheren Studien steht [6].

Im Vergleich zu anderen deutschen Studien lag die Sterblichkeit in unserer Kohorte mit 2,6 % im Durchschnitt [19, 20]. Die Risikostratifizierung und Prognosevorhersage bei akut vergifteten Patienten stellt eine Herausforderung in Zeiten mangelnder intensivmedizinischer Ressourcen dar. Nicht nur Intensivbetten sind im klinischen Alltag limitiert. Insbesondere in Pandemiezeiten ist uns bewusst, dass der Bettplatz mit technischer Ausstattung sowie das pflegerische und ärztliche Personal vorhanden sein muss, um die Patient:innen adäquat zu behandeln. Vergiftungspatienten werden mittels intensivmedizinischem Monitoring und therapeutischen Interventionen bis hin zum Organersatzverfahren aufwändig therapiert und binden somit wertvolles Personal. Wenn auch die Intoxikationspatienten im Mittel < 72 h auf der Intensivstation verbleiben, so werden materielle und personelle Ressourcen verbraucht, die an anderer Stelle fehlen. Der Prävention von Intoxikationen kommt deshalb auch in diesem Zusammenhang eine besondere Bedeutung zu. Zudem sind weitere Studien zur Pharmakodynamik der verschiedenen Toxine sowie zur Effizienz der primären und sekundären Toxinelimination erforderlich, um Risikopatienten zu identifizieren und die Notwendigkeit einer Behandlung auf der Intensivstation zu beurteilen [20].

Einzelne Limitationen der vorliegenden Studie sind zu erwähnen: Erstens wurden die Datenanalysen anhand der Kohorte eines einzelnen deutschen Zentrums durchgeführt, was eine Verallgemeinerbarkeit der Ergebnisse erschwert. Außerdem ist diese Studie durch ihr retrospektives Design begrenzt. Schließlich kann ein Informationsbias nicht vollständig vermieden werden, da einige Parameter, wie z. B. die Ursache der Intoxikation oder die Interventionsmaßnahmen, nicht für alle Patienten verfügbar waren.

Die in unsere Studie eingeschlossenen Patienten wurden im Rahmen ihres stationären Aufenthalts einer psychiatrischen Beurteilung sowie dem Angebot einer Anbindung zugeführt. Da eine psychiatrische/psychotherapeutische Weiterbehandlung jedoch teilweise in externen Versorgungsstrukturen erfolgte, liegen uns hierzu keine detaillierten Daten für die gesamte Kohorte vor. Dennoch kommt der poststationären Weiterbehandlung in unserem Kollektiv eine herausragende Bedeutung zu. In einer norwegischen Studie zu Vergiftungspatienten wurde beispielsweise gezeigt, dass ca. ein Drittel der Patienten nach einer Vergiftung keine weitere Anschlussbehandlung erhielt [22]. Die anderen zwei Drittel fanden Zuwendung im Rahmen des sog. Opioid-substitution-treatment(OST)-Programms und regelmäßiger Hausarztbesuch. In weiteren Studien aus Spanien und Australien wird belegt, dass Patienten, die an einem OST-Programm teilnehmen, geringere Mortalitätsraten aufweisen als Patient:innen ohne Follow-up-Behandlung [4, 21]. Spezifische Möglichkeiten für Anschlussbehandlungen sollten etabliert werden, um Umgang mit Medikamenten zu schulen und langfristig die Komorbidität und Mortalität zu senken.

Zusammenfassend lässt sich feststellen, dass unsere Studie einen wichtigen Überblick bietet, indem sie demographische und klinische Aspekte von akut vergifteten Patienten aufzeigt und mehr als 1000 Patienten über einen Zeitraum von 20 Jahren umfasst. Unsere Studie ergänzt daher nicht nur die Ergebnisse früherer Studien, sondern erweitert diese, indem sie ein größeres Patientenkollektiv analysiert und Vergiftungsmotive und Interventionsmaßnahmen im Detail untersucht. Die Notwendigkeit einer Intensivtherapie bei Intoxikationspatienten ist ein weltweit bekanntes Problem. Während epidemiologische Merkmale, wie Alter und Geschlecht, konstant bleiben, zeigt unsere Studie Veränderungen bei Verhaltensaspekten wie verwendeten Substanzen und Interventionsmaßnahmen über die letzten Jahre. Anhand der in unserer Studie dargestellten epidemiologischen und demographischen Charakteristika der verschiedenen Geschlechter- und Altersgruppen sowie der Prävalenz verschiedener Kontexte der Intoxikation wird deutlich, dass Intoxikationen, insbesondere der akzidentelle Gebrauch, der Substanzabusus sowie Suizidversuche in allen Altersgruppen manifest werden können und eine generationsübergreifende Prävention notwendig ist. Insbesondere männliche Patienten können aufgrund der Häufung von akzidentellem Gebrauch und Substanzabusus in dieser Geschlechtergruppe von einer gezielten Prävention profitieren, wie unsere Studie belegt. Das weibliche Geschlecht hingegen ist mit einem höheren Risiko eines Suizidversuchs assoziiert. Auch hebt unsere Studie hervor, dass insbesondere Patienten mit psychiatrischen Vorerkrankungen hinsichtlich einer Intoxikation gefährdet sind und ein vulnerables Patientenkollektiv darstellen, sodass auch hier präventive Maßnahmen verfügbar sein sollten.

## Fazit für die Praxis

- Vergiftungen stellen einen relevanten Anteil aller Aufnahmen auf die Intensivstation dar.
- Antidepressiva repräsentieren die am häufigsten verwendeten medikamentösen Substanzen bei Patienten mit einer akuten Vergiftung.
- Ältere Patienten weisen häufiger Vergiftungen mit Benzodiazepinen auf.
- Die Prävalenz von Multiorganversagen sowie die Mortalität bei akut intoxikierten Patienten sind gering.

## Abkürzungen

ACT: „Activated charcoal treatment“ (Behandlung mit Aktivkohle)
CO: Kohlenstoffmonoxid
ICCA: IntelliSpace Critical Care and Anaesthesia (Philipps, Inc., Hamburg, Deutschland)
ICD-10: „International statistical classification of diseases and related health problems, 10th revision“
NSAR: Nichtsteroidale Antirheumatika
WHO: World Health Organization
